# Redox dynamics in seeds of *Acer* spp: unraveling adaptation strategies of different seed categories

**DOI:** 10.3389/fpls.2024.1430695

**Published:** 2024-07-24

**Authors:** Hanna Fuchs, Aleksandra M. Staszak, Paola A. Vargas, Mariam Sahrawy, Antonio J. Serrato, Marcin K. Dyderski, Ewelina A. Klupczyńska, Paweł Głodowicz, Katarzyna Rolle, Ewelina Ratajczak

**Affiliations:** ^1^ Institute of Dendrology, Polish Academy of Sciences, Kórnik, Poland; ^2^ Laboratory of Plant Physiology, Department of Plant Biology and Ecology Faculty of Biology, University of Białystok, Białystok, Poland; ^3^ Department of Biochemistry, Cell and Molecular Biology of Plants, Estación Experimental del Zaidín, Consejo Superior de Investigaciones Científicas (CSIC), Granada, Spain; ^4^ Institute of Bioorganic Chemistry, Polish Academy of Sciences, Poznań, Poland

**Keywords:** seed physiology, *Trx-h1* regulation, metabolic adaptation, environmental cues, protein redox targets, seed viability

## Abstract

**Background:**

Seeds of woody plant species, such as those in the *Acer genus* like Norway maple (*Acer platanoides* L.) and sycamore (*Acer pseudoplatanus* L.), exhibit unique physiological traits and responses to environmental stress. Thioredoxins (Trxs) play a central role in the redox regulation of cells, interacting with other redox-active proteins such as peroxiredoxins (Prxs), and contributing to plant growth, development, and responses to biotic and abiotic stresses. However, there is limited understanding of potential variations in this system between seeds categorized as recalcitrant and orthodox, which could provide insights into adaptive strategies.

**Methods:**

Using proteomic analysis and DDA methods we investigated the Trx-h1 target proteins in seed axes. We complemented the results of the proteomic analysis with gene expression analysis of the *Trx-h*1, 1-*Cys-Prx*, and *TrxR NTRA* genes in the embryonic axes of maturing, mature, and stored seeds from two Acer species.

**Results and discussion:**

The expression of *Trx-h1* and *TrxR NTRA* throughout seed maturation in both species was low. The expression of 1-*Cys-Prx* remained relatively stable throughout seed maturation. In stored seeds, the expression levels were minimal, with slightly higher levels in sycamore seeds, which may confirm that recalcitrant seeds remain metabolically active during storage. A library of 289 proteins interacting with Trx-h1 was constructed, comprising 68 from Norway maple and 221 from sycamore, with distinct profiles in each seed category. Recalcitrant seed axes displayed a wide array of metabolic, stress response, and signaling proteins, suggesting sustained metabolic activity during storage and the need to address oxidative stress. Conversely, the orthodox seed axes presented a protein profile, reflecting efficient metabolic shutdown, which contributes to their extended viability. The results of the study provide new insights into seed viability and storage longevity mechanisms. They enhance the understanding of seed biology and lay the foundation for further evolutionary research on seeds of different categories.

## 1 Introduction

Humanity is facing new challenges in the areas of agriculture, forestry, and environmental protection due to the acceleration of global environmental changes (Díaz et al., 2006; Dyderski et al., 2018). Longer and more frequent droughts, unusual weather patterns, and increasing temperatures may lead to changes in ecosystems and potential species migration or extinction (Dyderski et al., 2018; Thurm et al., 2018; McDowell et al., 2020).

Seeds are a crucial component of ecosystems and play an important role in determining their composition and structure. Climate change can have substantial impacts on seed production, germination, and survival and can ultimately influence ecosystem dynamics (Baraloto et al., 2005) and crop production (Ghassemi-Golezani et al., 2009, 2015; Han et al., 2009). Therefore, appropriate seed storage is vital not only for economic and social reasons but also for environmental conservation (Hay and Probert, 2013; Leprince et al., 2016). Furthermore, seed aging is an important issue that affects gene banks. The viability of dry-stored seeds gradually decreases during the aging process, which is reflected by characteristics such as delayed germination, poor seedling establishment, or complete failure to germinate. However, seed traits receive insufficient attention in community ecology studies (Duncan et al., 2019).

The mechanisms underlying the regulation of desiccation tolerance at the molecular and genetic levels are still widely debated. Various factors can impact seed viability during storage, such as elevated reactive oxygen species (ROS) production, reduced antioxidant activity, and membrane degradation (Ratajczak et al., 2019c). ROS can play two roles in seeds: signaling molecules and toxic molecules that cause oxidative stress and ultimately aging (El-Maarouf-Bouteau and Bailly, 2008; Wojtyla et al., 2016; Małecka et al., 2021; Li et al., 2022). Alterations in the redox state of seeds can control various metabolic processes. Maintaining the cellular redox state is therefore crucial for optimal cell function and plays a critical role in regulating various signaling pathways to appropriately respond to environmental and developmental changes (Considine and Foyer, 2014; Smolikova et al., 2021).

The multigenic thioredoxin (Trx) family is believed to regulate the redox state in seed cells. The Trx *f*, *m*, *x*, *y*, and *z* isoforms are involved in chloroplast redox state regulation; the *o* isoform is found in mitochondria; and the *h* isoform is found in cytosol (reviewed in detail in Gelhaye et al., 2005; Mata-Pérez and Spoel, 2019). Trxs are most abundant in mature seeds and in the nucleus of cells under oxidative stress (Serrato and Cejudo, 2003). These Trxs are crucial for the germination process by reducing the amount of storage proteins and mobilizing lipids (Kobrehel et al., 1992; Wong et al., 1995; Babazadeh et al., 2012). Trxs may also be part of the antioxidative system due to their increased accumulation during the late stages of seed development and germination under oxidative stress (Serrato and Cejudo, 2003). Trxs play a central role in the redox regulation of cells by interacting with other redox-active proteins, such as peroxiredoxins (Prxs), and participating in plant growth, development, and response to biotic and abiotic stresses (Cejudo et al., 2012; Sevilla et al., 2015). In addition to interacting with Prxs, Trxs can also interact with other proteins involved in redox balance, such as Prx reductases and glutathione peroxidases (Meyer et al., 2008). Trxs act as electron donors for ribonucleotide reductase, a key enzyme in DNA synthesis, and for the enzyme methionine sulfoxide reductase, which is involved in the repair of oxidized proteins (Lee et al., 2013). In the Calvin cycle, Trxs reduce disulfide bonds in chloroplast proteins, enabling their activation under light conditions (Buchanan et al., 2002; Yokochi et al., 2021). Furthermore, Trxs regulate the activity of several enzymes in the tricarboxylic acid (TCA) cycle in plant mitochondria by modulating the redox state of their active sites (Daloso et al., 2015).

The Norway maple (*Acer platanoides* L.) and the sycamore (*Acer pseudoplatanus* L.) are both members of the same genus and are important components of temperate broadleaved forests (Ellenberg, 1988) maples are also widely used as ornamental trees (Oravec et al., 2023). Nevertheless, their seeds differ in desiccation tolerance and the duration of physiological embryo dormancy. Norway maple seeds are considered orthodox and can be safely dried for storage purposes. In contrast, sycamore seeds are sensitive to desiccation and will perish if their moisture content falls below 20%, placing them in the recalcitrant category (Hong and Ellis, 1990; Dickie et al., 1991). The physiology of Norway maple and sycamore seeds has already been well studied and documented in the literature (Daws et al., 2006; Pukacka and Ratajczak, 2007b; Pawłowski, 2009; Kalemba and Ratajczak, 2018; Ratajczak et al., 2019a; Plitta-Michalak et al., 2022). Therefore, the seeds of these two species serve as valuable models for research on the seed categories.

Our study builds upon previous research concerning the crucial role of redox regulation during key seed stages, including ripening, desiccation, and germination. Earlier investigations (Ratajczak et al., 2019a, 2019; Alipour et al., 2020; Kalemba et al., 2021) underscored the dynamic nature of redox regulation in seeds, highlighting its potential important impact on seed viability. In particular, Ratajczak et al. (2019a) demonstrated the importance of Prx proteins in regulating the redox state and viability of *Acer* seeds. Recent findings reported by Alipour et al. (2023), revealed heightened levels of redox control activity in sycamore seeds compared to Norway maple seeds.

Nevertheless, there is limited understanding of the potential differences in this system between recalcitrant and orthodox seeds, and these differences could shed light on adaptive strategies. To address this gap, we utilized embryonic axes from Norway maple and sycamore seeds, which were established as model systems for investigating tree seed biology. The aim of our study was to improve the understanding of seed tolerance to dehydration based on seed category. We conducted the first attempt to identify target proteins for Trxs in tree seeds via integrated proteomic studies involving the analysis of *Trx-h1* transcript level and that of two genes encoding proteins involved in this redox system, *1-Cys-Prx* and *TrxR NTRA*, via quantitative PCR (qPCR) in mature and stored seeds. Additionally, we confirmed the presence of the Trx-h1 protein in the studied seeds through Western blot analysis. Ultimately, our results contribute to a deeper understanding of the adaptive mechanisms inherent in different seed categories.

## 2 Materials and methods

### 2.1 Plant material collection

Norway maple (orthodox) and sycamore (recalcitrant) mature seeds were collected in 2020 from individual trees growing in Kórnik Arboretum (western Poland, 52°24′37′′N, 17°09′515′′E) for Trx-h1 target analysis. The seeds were then dehydrated to a moisture content of 10%, placed in plastic containers, and stored at 4°C. Seeds for transcript level analysis and Western blotting were collected from the same individual trees weekly during maturation, beginning from 11th for sycamore and 14th week after flowering (WAF) for Norway maple, over a period of 10 weeks from July until maturation in September. The isolated embryonic axes were immediately frozen in liquid nitrogen and stored at -80°C until analyses. The aging, mature seeds used for transcript level analysis where collected form the same trees, and were stored under typical conditions for the respective species (Suszka et al., 1996). We chose seeds stored for 2–5 years. Prior to analyses seed axes of stored seeds where imbibed in water overnight. Isolated seed axes were used for all analyses.

### 2.2 Mutagenesis, heterologous expression and purification of Trx *h1* from *Pisum sativum*

The *Pisum sativum* cDNA encoding Trx *h1* was amplified via PCR and subsequently cloned and inserted into the pET-28b expression vector, which translationally fused a His-tag to the N-terminal end of the protein. Next, Trx *h1* was PCR mutagenized by substituting the resolving cysteine residue, the second residue from the active site, with a serine residue (hereafter His-CXXS). The oligonucleotides used for cloning and site-directed mutagenesis were obtained from the publication by Serrato et al. (2018).

The resulting plasmid was purified following the MiniPrep (Nzytech, Lisbon, Portugal) protocol, verified by sequencing, and finally used to transform *Escherichia coli* BL21. Protein expression was induced with 0.4 mM isopropyl β-d-1-thiogalactopyranoside (IPTG) for 4 h when the bacterial culture reached a cell density at DO_600_ of 0.4. The recombinant protein was purified by coaffinity chromatography according to the manufacturer’s instructions (HiTrap Talon, Cytiva, Marlborough, MA, USA). The His-CXXS protein was then reduced by incubating with 50 mM dithiothreitol (DTT) for 20 min at RT. Excess DTT was further removed using 7K MWCO Zeba Spin Desalting Columns (Thermo Fisher Scientific, Waltham, MA, USA).

### 2.3 Binding of Trx *h1* to target proteins

#### 2.3.1 Proteins extraction

The embryonic axes of Norway maple and sycamore embryonic axes were homogenized in a cooled mortar with liquid nitrogen, using 200 mg per sample (three biological replicates). After that, the resulting homogenate was suspended in extraction buffer containing 50 mM Tris-HCl (pH 7.8), 10 mM KCl, 20% glycerol, and a complete (TM) protease inhibitor cocktail (Roche, Basel, Switzerland) to extract the soluble protein. The protein content was measured using the Bradford method (Bradford, 1976).

#### 2.3.2 Targets capture and purification

To capture the Trx-h1 target, protein extracts from embryonic axes were incubated with His-CXXS for 30 minutes at room temperature (RT) under gentle agitation. After incubation, the CXXS-target complexes were purified using Dynabeads pull-down (Invitrogen, Waltham, MA, USA), which binds His-tagged proteins, following the manufacturer’s instructions. Next, the purified complexes were incubated with 50 mM DTT for 30 min at RT to reduce the disulfide bridge and separate the targets from His-CXXS. Finally, DTT was removed by spin dialysis (Zeba™ Spin Desalting Columns, 7K MWCO, Thermo Fisher Scientific) with a column equilibrated with Dynabeads binding buffer. Finally, to isolate the Trx-h1 targets, Dynabeads were again used for pull-down, but the wash fraction, where the target was present, was removed instead of the elution fraction, which contained only His-CXXS. The targets were then incubated overnight with 15% trichloroacetic acid at -20°C and centrifuged at 4°C for 20 min at 15000 × *g*. After isolating the targeted protein, we performed Western blotting with a Trx-h1 antibody (rabbit) at a 1:5000 dilution, to detect monomers, dimers, and complexes (probe without DTT), in the presence a negative control probe with the isolated protein and DTT. The pellet was washed twice with 80% (v/v) ice-cold acetone before liquid chromatography-tandem mass spectrometry (LC −MS/MS) analysis.

#### 2.3.3 LC −MS/MS analysis of proteins

The precipitated target proteins were analyzed according to previous methods described by Torres-Romero et al. (2022). To achieve a final concentration of 5 mM, dithiothreitol was added to the samples, followed by a 30-minute incubation at 60°C. Next, iodoacetamide was added to a final concentration of 10 mM, and the samples were incubated for 30 minutes at RT, protected from light. Enzymatic digestion was performed by incubating the solution with trypsin at a 1:40 ratio (trypsin:protein) overnight at 37°C. Post-digestion, 1 µg of protein was subjected to analysis using a Tandem Quadrupole Time-of-Flight mass spectrometer (AB/Sciex TripleTOF5600 Plus) linked to a Nanospray III Ion Source (AB/Sciex) and a nano-HPLC (EKsigent Ultra 2D). Peptide separation involved the removal of impurities on an isocratic pre-column (C18 PepMap100 column NAN75–15-03-C18-PM, Thermo Fisher Scientific) with a solvent consisting of 0.1% formic acid and 5% (v/v) acetonitrile, delivered at a flow rate of 3 µl min−1 for 10 minutes. The peptides were then transferred to the analytical column through an integrated electrospray emitter (New Objective PicoFrit column, 75 µm internal diameter × 250 mm, packed with Reprosil-PUR 3 µm) and separated using a linear gradient of solvent B from 5% to 35% over 60 minutes, at a flow rate of 250 nl min−1. Solvent A was composed of 0.1% (v/v) formic acid, and solvent B was acetonitrile with 0.1% (v/v) formic acid. The ion source was set with parameters: ISVF=2600, GS1 = 20, CUR=25. Data acquisition was carried out using the DDA method, starting with a high-resolution TOF-MS scan over a mass range of 400–1250 m/z, followed by MS/MS scans of 50 ion candidates per cycle, covering a mass range of 230–1500 m/z with dynamic background subtraction, operating in high-sensitivity mode. Ion accumulation times were set to 250 ms for MS and 65 ms for MS/MS. Protein identification was performed using ProteinPilot v5.0.1 software (Sciex) with the Paragon algorithm and a database of the *Acer yangbiense* in FASTA format, combined with the Sciex contaminant database from www.uniprot.org.

### 2.4 Western blot

Protein samples (20 µg) were loaded onto a 12% SDS −PAGE gel. The separated proteins were transferred to a polyvinylidene fluoride membrane (ImmobilonTM-P; Merck Millipore, Burlington, MA, USA) at 350 mA for 1 h using a Mini Trans-BlotCell (Bio-Rad, Hercules, CA, USA). The membranes were blocked with 1% bovine serum albumin (BSA) and incubated with antibodies against Trx-h1 at a 1:5000 dilution (kindly provided by Prof. Bob B. Buchanan; produced at the University of California at Berkeley). The secondary antibody, goat anti-rabbit IgG conjugated with alkaline phosphatase (Sigma −Aldrich, St. Louis, MO, USA), was used at a 1:3000 dilution to visualize protein bands by reaction with 5-bromo-4-chloro-3-indolyl phosphate/nitroblue tetrazolium (BCIP/NBT, Sigma −Aldrich) as a substrate. The results obtained from three experiments were digitized and statistically tested for significant differences using one-way ANOVA.

### 2.5 Native PAGE superoxide dismutase activity

SOD isoenzymes in Acer seeds were identified according to Beauchamp and Fridovich (1971), based on the inhibition of SOD activity in the reduction of NBT to formazan blue by O ^2 -^ radicals. Proteins were separated via electrophoresis with 10–15 mA/gel for 30 minutes, followed by 25 mA/gel (Bio-Rad, Hercules, CA, USA). After separation, the gels were immersed in KCN and H_2_O_2_ to identify SOD isoenzymes as inhibitors: Cu/Zn-SOD is inhibited by KCN and H_2_O_2_, Fe-SOD is inhibited by H_2_O_2_, and Mn-SOD is resistant to both inhibitors.

### 2.6 RNA isolation and qPCR analysis

Total RNA from seed axes (three replicates of 10 embryonic axes per sample) was extracted using an RNeasy PowerPlant Kit (Qiagen, Hilde, Germany) according to the manufacturer’s instructions. First-strand cDNA was synthesized from total RNA using a Transcriptor First Strand cDNA Synthesis Kit (Roche). cDNA was subsequently subjected to qPCR, which was run on a CFX Connect Real-Time PCR Detection System (Bio-Rad) and carried out on 96-well plates. Specific primers were designed based on the *Arabidopsis* reference genome sequence. A list of primer sequences is available in **Supplementary Table 1**. Three biological replicates and 3 technical replicates of each transcript were analyzed. The 2^-ΔΔCt^ method was used as a relative quantification strategy for qPCR data analysis (Livak and Schmittgen, 2001). Elongation factor Tu family protein (*EF1A*) was chosen as the housekeeping gene, while the reference sample was derived from embryonic axes of mature seeds from the latest harvest. Subsequently, the data were analyzed using GraphPad Prism v. 8.4.3 for Windows (GraphPad Software, Boston, MA USA, www.graphpad.com). The Shapiro test was conducted to assess whether the data obtained via the 2^-ΔΔCt^ method followed a normal distribution, assuming a p value threshold of 0.05. If the data did not follow a normal distribution, they were transformed using the natural logarithm. Changes in expression were analyzed using two-way ANOVA (levels: time, species, time x species). *Post-hoc* analysis was performed using Tukey’s posteriori test. Graphical presentation of the results was generated using GraphPad Prism v. 8.4.3 for Windows.

### 2.7 Oxygen consumption rate and extracellular acidification rate measurements of the seed axes

To check the viability of the embryonic axes, we assessed their respiratory activity using a Seahorse XF HS Mini Analyzer (Agilent Technologies, Inc., Santa Clara, CA, USA) according to the methods of Sew et al. (2013). Seed axes (two replicates of three seed axes per well) were placed in eight-well plates and surface sterilized by soaking for 7 min in 12.5% (w/v) NaClO and 0.1% (v/v) Tween 20, followed by two washing steps with distilled H_2_O. Subsequently, the wells were filled with 200 μL of respiration medium (5 mM KH_2_PO_4_, 10 mM TES, 10 mM NaCl, 2 mM MgSO_4_, pH 7.2) and loaded into the plate reader after the calibration steps using Seahorse XF Calibrant Solution (Agilent Technologies). The oxygen concentrations were determined by 75 cycles of mixing (3 min) and measurement (3 min). The OCR and ECAR of the seed axes were analyzed using Wave v. 2.6.1 software (Agilent Technologies). The data were exported to GraphPad, where subsequent statistical analysis was conducted. The normality of the data was assessed using the Shapiro −Wilk test, followed by two-way ANOVA (levels: time, species, time x species). Tukey’s posteriori test was employed as a *post hoc* test. Next, to assess the respiration phenotype, we assessed the relationship between OCR and ECAR using a Pearson correlation coefficient analysis.

## 3 Results and discussion

**Figure 1:**
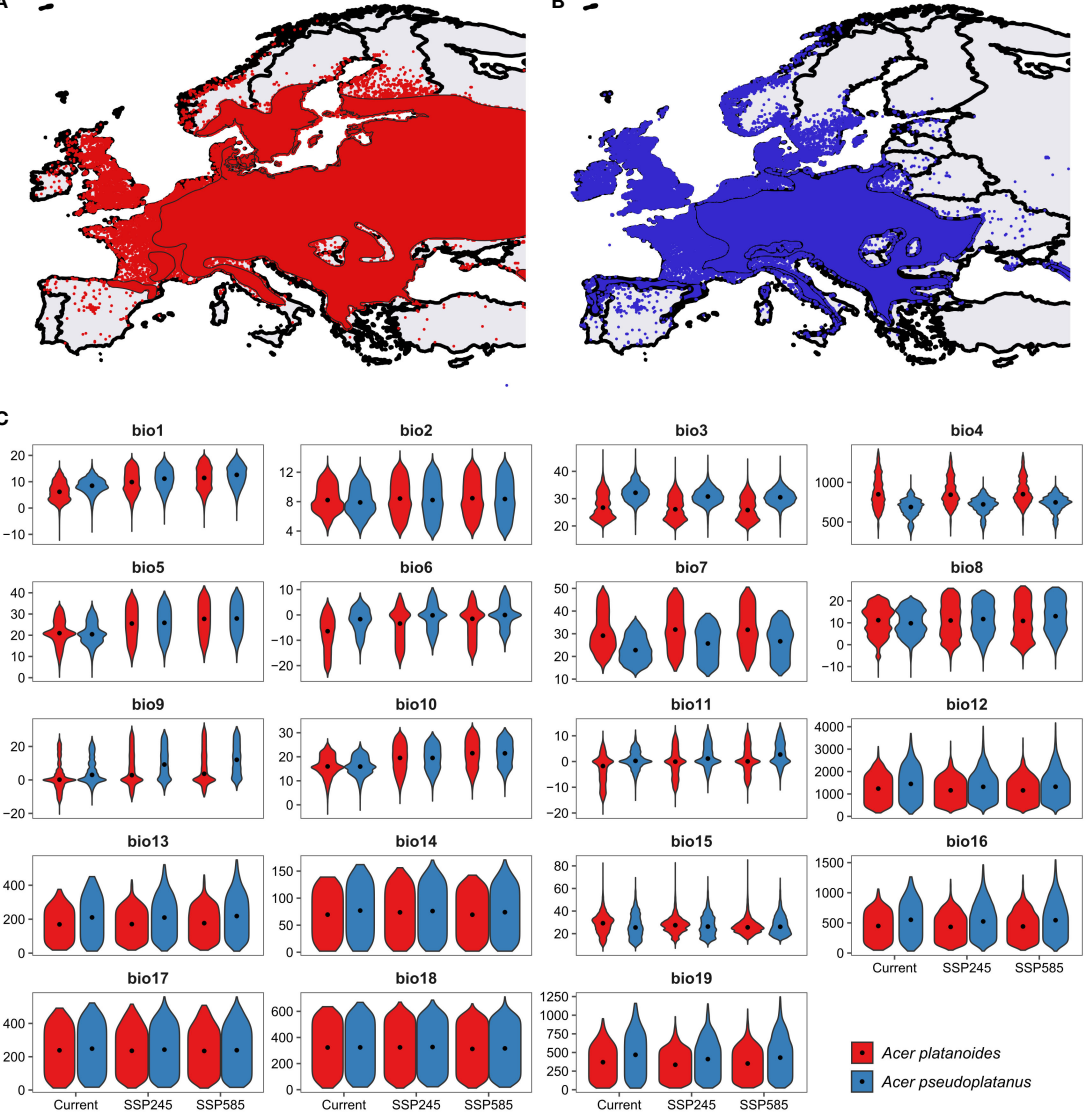
Distribution of **(A)** Norway maple and **(B)** sycamore, **(C)** polygons represent range described by Caudullo et al. (2017) while points – distribution from GBIF (2023), and distributions of 19 bioclimatic variables. bio1 – Mean annual temperature (°C), bio2 – Mean diurnal temperature range (°C), bio3 – Isothermality (dimensionless), bio4 – Temperature seasonality (standard deviations*100), bio5 – Mean maximum temperature of the warmest month (°C), bio6 – Mean minimum temperature of the coldest month (°C), bio7 – Annual range of temperature (°C), bio8 – Mean temperatures of the wettest quarter (°C), bio9 – Mean temperatures of the driest quarter (°C), bio10 – Mean temperatures of the warmest quarter (°C), bio11 – Mean temperatures of the coldest quarter (°C), bio12 – Mean annual precipitation (mm), bio13 – Precipitation of the wettest month (mm), bio14 – Precipitation of the driest month (mm), bio15 – Precipitation seasonality (dimensionless), bio16 – Mean precipitation of the wettest quarter (mm), bio17 – Mean precipitation of the driest quarter (mm), bio18 – Mean precipitation of the warmest quarter (mm), bio19 – Mean precipitation of the coldest quarter (mm). Specific values are presented in **Supplementary Table 2**. Violins represent distributions of bioclimatic variables within ranges, dots – median values.

Norway maple and sycamore, both of which belong to the genus *Acer*, are ecologically important and exhibit varying sensitivities to environmental stressors, which are further exacerbated by ongoing climate change (**Figure 1**). Notably, Norway maple begins flowering approximately three weeks earlier than sycamore does, while the morphogenesis of embryos commences earlier in sycamore. Consequently, both species yield mature seeds that are shed simultaneously during autumn (Pukacka, 1998). These species do not differ in terms of seedling functional traits, biomass allocation, or relative growth rate (Dyderski and Jagodziński, 2019; Fanal et al., 2022), but they do differ in terms of their soil moisture requirements. Ellenberg’s ecological indicator value for sycamore is 6 (between fresh and humid but not wet soils), while that for Norway maple is not specified, indicating a wide range of moisture requirements (Ellenberg and Leuschner, 2010). A similar range of drought resistance was provided by Caudullo and de Rigo (2016) and Pasta et al. (2016) for these species; however, these authors reported greater habitat suitability of Norway maple in the eastern part of the EU and for sycamore in the central belt of the EU from the British Isles through France and Austria to Romania and the Balkan Peninsula. Analysis of bioclimatic variables within the ranges of the two studied *Acer* species revealed differences between species: sycamore currently occurs in areas with 2.0°C higher mean annual temperature, 225 mm greater mean annual precipitation, and lower temperature and precipitation seasonality (**Figure 1**; **Supplementary Table 2**). However, the difference between precipitation in the warmest quarter was statistically nonsignificant (p=0.89), indicating similar water availability in the period with the highest drought probability. These patterns will be similar under both intermediate (‘Middle of the Road’) and worst-case climate (‘Fossil-fuel development) change scenarios, representing the shared socioeconomic pathways intermediate and worst-case, respectively (Riahi et al., 2017). Dyderski et al. (*under review*) predicted contractions of 24.1% and 47.1% of the current range for intermediate and worst-case, respectively. For Norway maple, the predicted contraction under those scenarios is 10.2% and 21.9%, respectively. For that reason, identifying the mechanisms responsible for drought adaptation is particularly important for conserving these species and maintaining their management.

Seeds from both *Acer* species exhibit dormancy, with germination spanning 12–20 weeks for Norway maple and 8–15 weeks for sycamore (Suszka et al., 2000). The distinct desiccation tolerance levels of these seeds render them ideal models for exploring various metabolic processes and alterations in cellular redox states during seed maturation, desiccation, and storage. Furthermore, our prior findings suggest that the embryonic axes of seeds from the genus *Acer* are particularly susceptible to oxidative alterations. This sensitivity underscores the crucial role of redox state regulation in maintaining seed viability and facilitating adaptation to changing environmental conditions (Pukacka and Ratajczak, 2007b; Ratajczak et al., 2019c). Thus, we conducted our studies on isolated seed axes.

### 3.1 Transcript level and Western blot analysis

Before analyzing the Trx-h1 target proteins in the embryonic axes of seeds, we performed transcript level analysis and Western blotting to confirm the presence of this protein in the seeds of the studied species. The expression levels of the *Trx-h1*, *TrxR NTRA* and 1-*Cys Prx* genes were assessed throughout seed development and storage for various durations in both orthodox and recalcitrant seeds.

**Figure 2:**
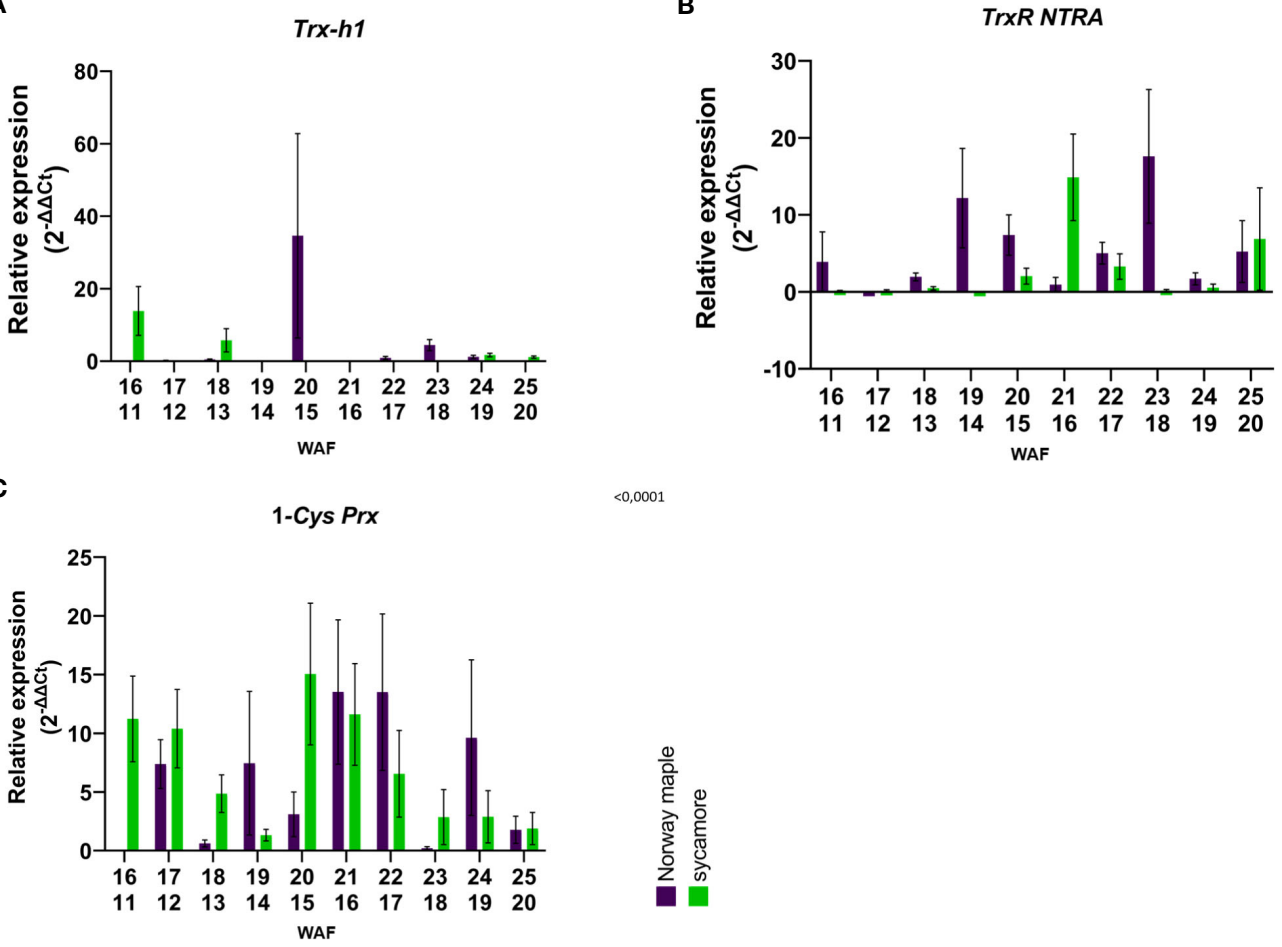
Relative expression patterns of **(A)** *Trx-h1*, **(B)** *TrxR NTRA*, and C) *1-Cys-Prx* in the axes of mature seeds of Norway maple (violet) and sycamore (green). The results represent the mean 2^-ΔΔCt^ values (Y axis) obtained from three replications and calculated relative to the housekeeping gene elongation factor Tu family protein (*EF1A*) and a reference sample representing embryonic axes from mature seeds of the last harvest term. The numbers on the x-axis represent the weeks after flowering (WAF) for Norway maple (above) and sycamore (below). Analysis of variance (ANOVA, factors time, species, time x species) was performed to test the statistical significance between tested species: For *Trx-h*1 and *TrxR NTRA* factor time x species was statistically significant P values <0.0001**** and 0.0264* respectively. For 1-*Cys-Prx* time P value 0.0064 **, species P value 0.7881, time x species P value 0.0386 *.

**Figure 3:**
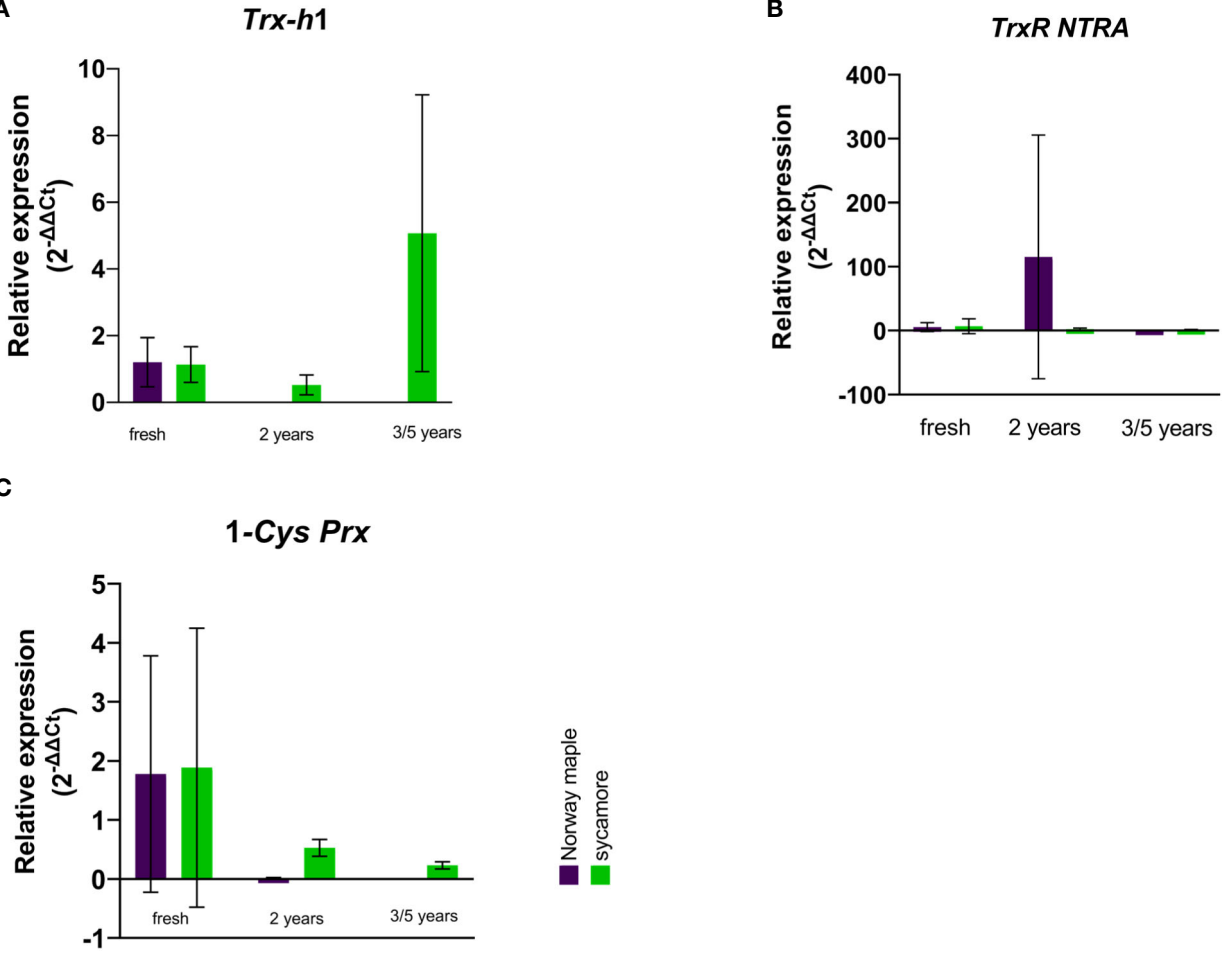
Relative expression patterns of **(A)** *Trx h*, **(B)** *TrxR NTRA*, and **(C)** *1-Cys Prx* in the axes of stored seeds of Norway maple (violet) and sycamore (green). The results represent the mean 2^-ΔΔCt^ values (Y axis) obtained from three replications and calculated relative to the housekeeping gene elongation factor Tu family protein (*EF1A*) and a reference sample representing embryonic axes from mature seeds of the last harvest term. Analysis of variance (ANOVA, factors time, species, time x species) was performed to test the statistical significance between tested species: no statistical significance was observed for *Trx-h1, Trx R NTRA*, for 1-*Cys-Prx* factor time was statistically significant P value 0.0093**.

In the embryonic axes of recalcitrant sycamore seeds during maturation, the expression of the *Trx-h1* gene reached its highest peak at 14th WAF. Subsequently, the expression decreased, exhibiting another peak only toward the end of the maturation period at 24 WAF. In the case of the Norway maple orthodox seeds, the transcript levels were below our detection threshold, with the only observed peak occurring at 14th WAF. Our results suggest that this gene exhibits distinct expression profiles in recalcitrant and orthodox seeds (**Figure 2A**). In the embryonic axes of recalcitrant seeds stored for 2 years, we observed low expression of the *Trx-h1* gene, with expression levels falling below detectable limits in seeds stored for longer periods. Conversely, in the embryonic axes of orthodox seeds, the expression level was below the threshold (**Figure 3A**). However, in the case of orthodox seeds, this lack of expression may indicate a state of deep dormancy and reduced metabolic demand; for recalcitrant seeds, this may suggest a loss of viability.

We observed a low level of *TrxR NTRA* transcript level in recalcitrant sycamore seed axes during maturation. Interestingly, two weeks prior to the increase in expression of the *Trx-h1* gene, a significant increase in expression of the *TrxR NTRA* gene was observed. On the other hand, in the embryonic axes of orthodox Norway maple seeds, the expression level of this gene remained low throughout the maturation period, falling below our detection limits from 19th WAF onward (**Figure 2B**). A modest level of expression in the embryonic axes of stored recalcitrant seeds was observed, and the level decreased over time. In the embryonic axes of orthodox seeds, we noted minimal expression in stored seeds (**Figure 3B**).

The expression of the 1-*Cys Prx* gene in the embryonic axes of recalcitrant seeds remained relatively stable throughout the entire maturation period, exhibiting a notable decrease near the end at 23th WAF. Conversely, in the orthodox seed axes, the expression of this gene was significantly lower than that in recalcitrant seed axes. Toward the end of maturation, from 20th WAF onward, the concentration fell below the detection threshold (**Figure 2C**). During storage, the expression of the 1-*Cys Prx* gene was minimal but significantly higher in sycamore maple seeds (**Figure 3C**).

Trxs are essential for the germination process in seeds, as they mobilize reserve materials by enhancing the solubility and susceptibility of storage proteins to proteolysis through reduction, deactivating disulfide proteins that inhibit specific amylases and proteases, thus facilitating the degradation of stored starch and proteins, and reductively activating individual enzymes that are crucial for germination (Kobrehel et al., 1992; Yano et al., 2001; Wong et al., 2004b; Alkhalfioui et al., 2007). The prominent peaks observed in the embryonic axes of sycamore seeds for these two genes could hypothetically indicate their importance in facilitating germination after a short period of dormancy, a trait typically associated with recalcitrant seeds.

TrxRs are a part of the NADPH-dependent thioredoxin system and they are highly conserved and distributed across cytoplasm, mitochondrion, chloroplast and nucleus (Serrato et al., 2004; Reichheld et al., 2005; Marchal et al., 2014). Their function involves managing the redox states of targeted proteins to uphold cellular ROS equilibrium. TrxR catalyzes the reduction of Trx in a process dependent on NADPH. Although, the role of TrxR NTRA in both plants and mammals has not been well understood, there is evidence that NTRA affects oxidative stress tolerance. *Arabidopsis* plants overexpressing NTRA showed increased tolerance to oxidative stress, evidenced by lower levels of toxic ROS molecules compared to wild-type and NTRA knock-out mutants. NTRA played a role in maintaining ROS homeostasis and reducing oxidative damage from various stresses. This ROS-regulatory function was particularly evident in NTRA overexpressing plants, which demonstrated greater drought stress tolerance (Cha et al., 2014).

1-*Cys Prx* is expressed in seeds in changing environments that cause oxidative stress (Dietz, 2011). 1-*Cys Prxs* alleviate mild oxidative stress and act as molecular chaperones during seed germination and plant development under severe conditions. It has been observed that overoxidation plays a crucial role in switching the function of 1-Cys-Prxs from that of peroxidases to that of molecular chaperones (Kim et al., 2011a). 1-Cys-Prx proteins are involved in desiccation tolerance during the late stages of seed maturation (Kim et al., 2011a; Ratajczak et al., 2019c) and plays a regulatory role in the release of seed dormancy (Kim et al., 2011a; Pawłowski and Staszak, 2016). Bykova et al. (2011) identified 1-Cys-Prx as a redox-sensitive protein characterized by the presence of reduced cysteine in both dormant and nondormant orthodox *Triticum aestivum* seeds. Previous Western blot analyses revealed the presence of 1-Cys-Prx in both *Acer* species. However, distinct patterns were observed in the redox gels. In Norway maple seeds, 1-Cys-Prx exhibited a redox-sensitive conformation and interaction. Conversely, in sycamore seeds, 1-Cys-Prx possibly exhibited less redox-dependent dynamic behavior (Ratajczak et al., 2019a). In our study, the axes of recalcitrant sycamore seeds presented higher expression of this gene than did the axes of orthodox Norway maple seeds, which may indicate their heightened sensitivity to stressful conditions. This, in turn, underscores the necessity for a greater presence of this protein, highlighting its crucial role in adapting to challenging environmental conditions and rapid germination.

The role of this system during seed maturation is not yet well understood. Most studies have been conducted on the later stages of maturation or during germination. Our research is a first step towards understanding the significance of this system for the maturation process of seeds from different categories.

**Figure 4:**
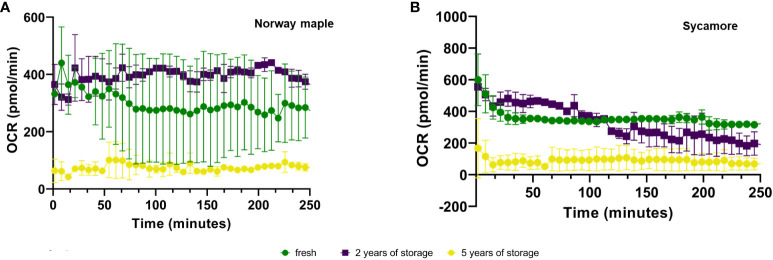
Mean ± SE oxygen consumption rate (OCR) values of the embryonic axes of Norway maple **(A)** and sycamore **(B)** seeds for fresh and stored seeds for 2 and 5 years. Analysis of variance (ANOVA) was performed for storage time, P value <0.0001 ***, as well as for storage period fresh P value <0,0001 ****, 2-year P value <0,0001 ****, 5-year P value <0,0001 ****.

**Figure 5:**
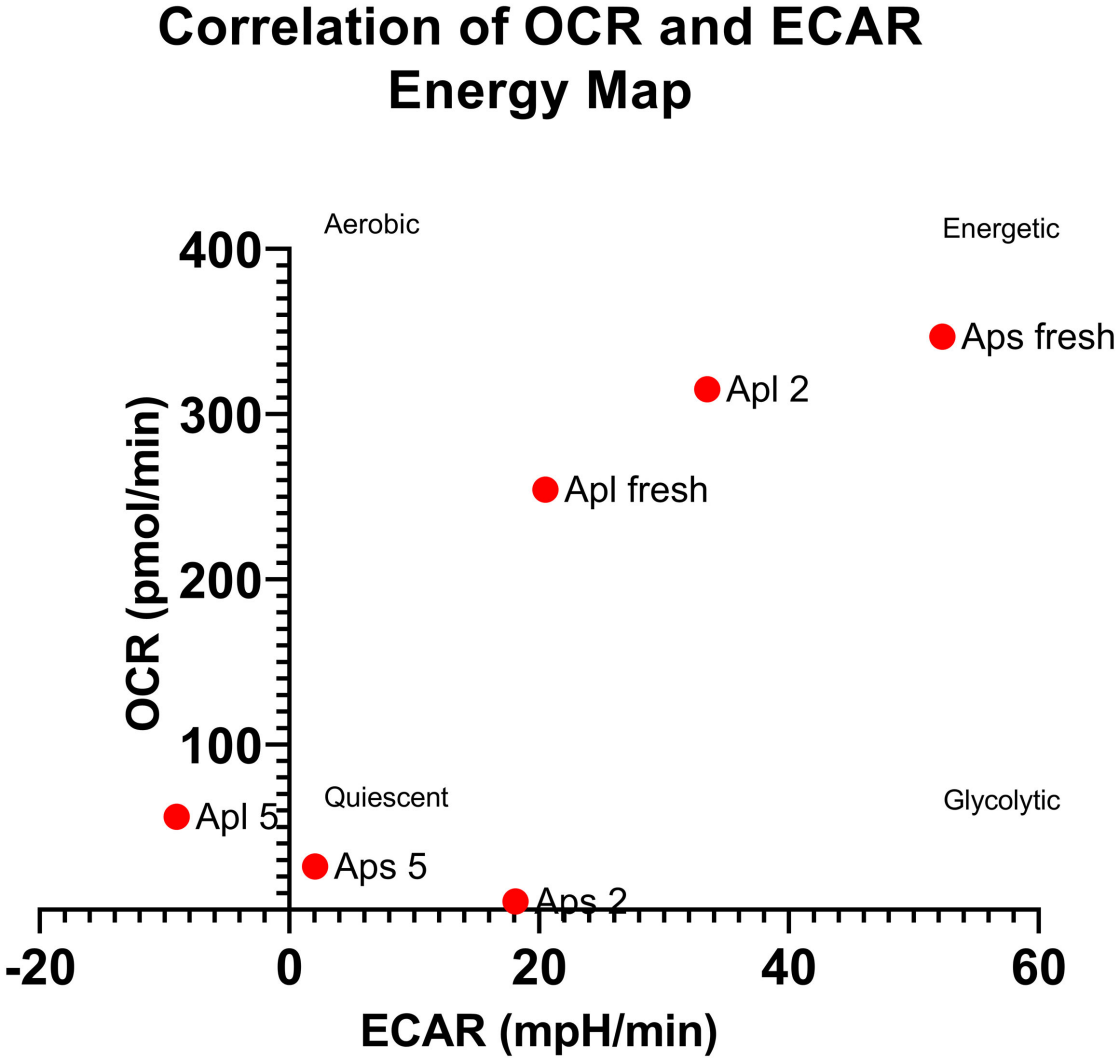
Relationship between the mean OCR and ECAR presented as an energy map, allowing conclusions about cell viability and energy phenotype (Zhang et al., 2012; Keuper et al., 2019; Vijayan et al., 2019). Apl-Norway maple, Aps-sycamore (fresh, 2-seed axes stored for two years, 5-seed axes stored for 5 years).

Expression analysis of the embryonic axes of stored seeds suggested that recalcitrant seeds remain metabolically active. Although the relative expression level of these genes was low, it was significantly greater than that in the embryonic axes of orthodox seeds. Over time, there was a declining trend in the expression levels, which may indicate a loss of viability. To assess the viability of these axes, we analyzed the OCRs and ECARs of the seeds. The results revealed that the OCR depends on both the species and the storage duration (**Figure 4**). Next, to determine the respiration phenotype, we assessed the relationship between OCR and ECAR, which enabled us to present OCR/ECAR ratio data as an energy map (**Figure 5**). The OCR/ECAR ratio allows for the evaluation of cell viability and energy phenotype (Zhang et al., 2012; Keuper et al., 2019; Vijayan et al., 2019). Moreover, Seahorse Analyser has been adapted to study respiration of plant tissues and organs (Sew et al., 2013; Fuchs et al., 2023). These results showed that the embryonic axes of Norway maple seeds maintained viability longer (**Figure 5**). However, in the case of sycamore, after only two years of storage, we observed a shift toward glycolysis in the metabolism of the embryonic axes (**Figure 5**). Therefore, results suggest that the decrease in transcript level in orthodox seeds was a consequence of metabolic slowdown and deep dormancy, while in recalcitrant seeds, the decrease in expression is due to loss of viability.

**Figure 6:**
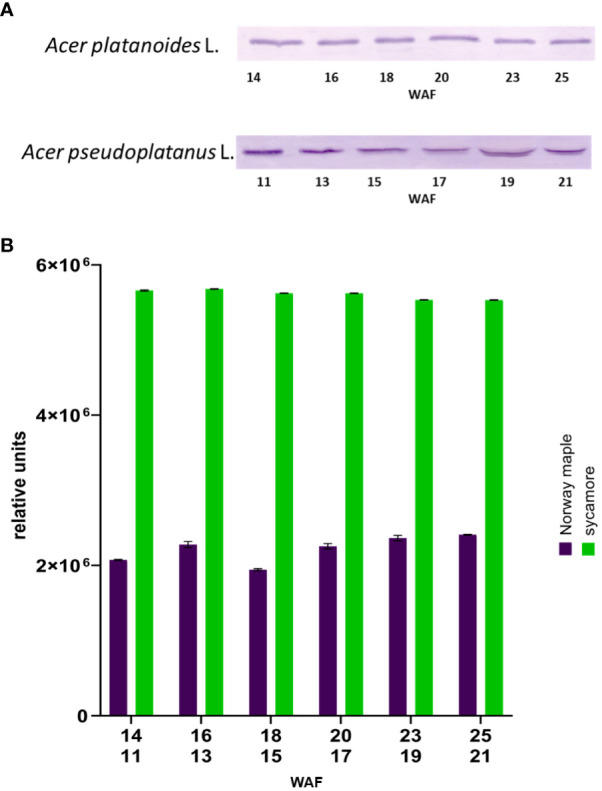
Western blot identification of Trx*h1* in embryonic axes of Norway maple and sycamore seeds during their development **(A)** image of immunodetection, **(B)** relative level of detected Trx-*h1* proteins in seed axes. WAF-weeks after flowering for Norway maple (above) and sycamore (below). Two-way analysis of variance (ANOVA) performed to test the statistical significance between tested species: P value for factor species, time and species x time <0.0001 ****.

The next step in confirming the presence of Trx-h1 in the seeds of the studied trees involved Western blot analysis. Immunodetection of Trx-h in the embryonic axes of Norway maple and sycamore seeds during development via Western blotting was performed using specific antibodies for Trx-h (**Figure 6**).

We examined the embryonic axes of both developing and mature seeds. Trx-h1 protein levels were greater in the embryonic axes of Norway maple seeds than in those of sycamore seeds. Trx-h1 protein levels remained constant throughout the maturation of these seeds. In the case of the embryonic axes of sycamore seeds, the level of Trx-h1 protein decreased during maturation. (**Supplementary Figure 1**).

### 3.2 Trx-h1 target protein assay

Proteomic techniques enable the identification of a multitude of potential Trx target proteins via tailored methodologies. Among these, the application of Trx mutant affinity chromatography has gained substantial prominence in target protein identification. This approach capitalizes on the capacity of a mutated Trx-h1 C/S complex, which features a nonfunctional active site cysteine, to capture target proteins through the establishment of a stable Trx-target complex.

This method is gaining popularity because it enables the identification of target proteins in model species (Alkhalfioui et al., 2007; Ueoka-Nakanishi et al., 2013; Yoshida et al., 2013; Mihara et al., 2017; Wu et al., 2021). However, there is still a lack of relevant information for woody plant species, which form incredibly important ecosystems that are greatly threatened by global climate change.

**Figure 7:**
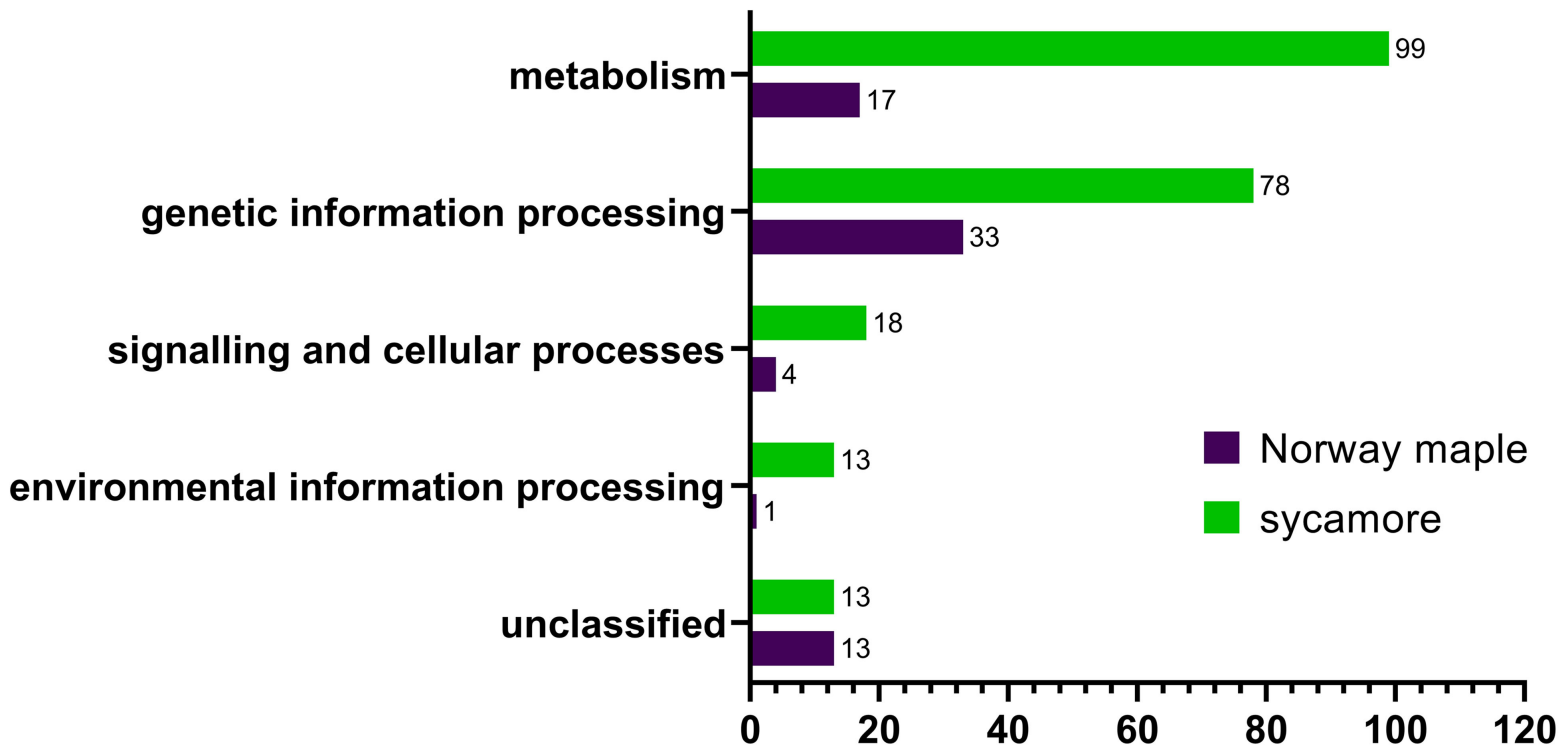
Categorization of Trx-*h1* target proteins in the embryonic axes of Norway maple and sycamore according to the KEGG database.

The cysteine to serine modification of Trx allowed us to separate proteins that are potentially regulated by the Trx-h1 complex from the intact soluble extract in orthodox Norway maple and recalcitrant sycamore seeds. Using DDA methods, a library of 289 proteins that form complexes with Trx-h1 was constructed (**Supplementary Table 3**), including 68 from Norway maple and 221 from sycamore. From a global perspective, these findings demonstrated significant differences in the proteome, which potentially falls under the control of redox regulation. The identified proteins belonged to various functional categories according to the KEGG database, such as cellular processes, genetic information processing, metabolism, and environmental information processes, as well as a group of unclassified proteins (**Figure 7**, **Table 1**, **Supplementary Table 3**). Due to the large number of identified proteins, we decided to focus our discussion in this paper only on a subset of proteins that appeared to be the most significant in terms of differences in adaptive strategies among the discussed seed categories. This subset was divided into subsections corresponding to their functions according to the KEGG database classification.

**Table 1:** The number of proteins identified in the embryonic axes of Norway maple and sycamore seeds, categorized based on the KEGG database.

Category	sycamore	Norway maple	Shared proteins	
Metabolism	99	17	8	Glutathione peroxidase Glutathione transferase Glyceraldehyde-3-phosphate dehydrogenase Non-specific lipid-transfer protein Nucleoside diphosphate kinase Phosphoglucomutase (alpha-D-glucose-1,6-bisphosphate-dependent) SERPIN domain-containing protein Thioredoxin-dependent peroxiredoxin
Genetic Information processing	78	33	8	60S acidic ribosomal protein P0 EF1_GNE domain-containing protein Elongation factor 1-alpha Peptidase A1 domain-containing protein SHSP domain-containing protein Tr-type G domain-containing protein WHy domain-containing protein AAA domain-containing protein
Signaling and cellular processes	18	4	2	Na_H_Exchanger domain-containing protein Actin
Environmental information processing	13	1	1	Superoxide dismutase [Cu-Zn]
Unclassified	13	13	0	–

In mature seed, to determine the presence of complexes of Trx with target proteins, we used protocol with and without DTT. After addition of DTT strongly visible was monomer of Trx*h1* (**Supplementary Figure 1**). We detect that number of Trx*h1* protein is high.

#### 3.2.1 Metabolism

We identified 99 and 17 proteins connected to metabolism in the sycamore and Norway maple proteomes, respectively. Among the entire set of identified proteins, only eight were common to both species (**Table 1**), emphasizing the stark dissimilarity in their metabolic protein repertoire. This discrepancy likely mirrors the varying metabolic demands and adaptations of recalcitrant and orthodox seeds in response to their respective ecological niches and storage capacities. Recalcitrant sycamore seeds are metabolically active and do not undergo a decrease in metabolism during storage, unlike orthodox seeds, whose metabolism is downregulated, which decreases their susceptibility to the effects of oxidative stress (Leprince et al., 1999; Wang et al., 2015; Hell et al., 2019).

The distinctive protein composition found exclusively in recalcitrant seeds appears to be intricately linked to pivotal metabolic processes, including ATP synthesis, the TCA cycle, pyruvate metabolism, and carbohydrate metabolism (**Supplementary Table 3**). Several subunits of ATP synthase have been identified in recalcitrant seeds; in contrast to the fact that they exhibit desiccation tolerance, scientists postulate that seeds must reduce their metabolic activity (Leprince et al., 2016).

Both mitochondrial and chloroplast ATPase subunits are subject to redox control by Trxs. Within plants, the chloroplast F_1_F_O_-ATPase is subject to intricate redox regulation, orchestrating the modulation of ATP hydrolytic activity through the reduction (activation) of a disulfide bond positioned within the γ subunit (Wang et al., 2011; Kim et al., 2011b). Our results suggest that the F_1_F_O_ alpha and beta subunits and the CF_1_ alpha subunit may constitute distinctive targets for interactions with Trxs in recalcitrant seeds, while the CF_O_ c subunit appears to play a comparable role in orthodox seeds. In recalcitrant seeds, the interplay between Trx and the alpha and beta subunits of ATPase may play a critical role in tailoring the activity of the enzyme to prolonged seed storage.

The TCA cycle plays a pivotal role within the metabolic network. This cycle oxidizes acetyl coenzyme A, which is generated through the catabolic breakdown of carbohydrates, amino acids, and fatty acids. Notably, TCA intermediates serve as integral components of biosynthetic processes, contributing to the synthesis of glucose, heme, lipids, and amino acids, among others. Housed within the mitochondrial matrix, the enzymes that orchestrate the TCA cycle form a supramolecular assembly, intimately interacting with mitochondrial membranes and serving as a crucial source of reducing power for the mitochondrial respiratory chain (Lushchak et al., 2014). In this study, specific enzymes involved in the TCA cycle were identified within the recalcitrant seed category (**Supplementary Table 3**). Importantly, a specific target of Trx exclusive to recalcitrant seeds, as identified in this investigation, is the enzyme malate dehydrogenase (MDH). Operating as a crucial component within the TCA cycle, MDH catalyzes the oxidation of malate. Several proteomics studies have suggested that mitochondrial MDH is a Trx target (Balmer et al., 2004; Marchand et al., 2010; Yoshida et al., 2013). The identification of MDH solely in stored recalcitrant seeds may indicate the potential role of its redox regulation in the maintenance of metabolic processes during seed storage and preservation. This observation might suggest that MDH could be involved in supporting essential biochemical reactions that contribute to seed viability and survival under the specific conditions of recalcitrant seed storage. As reported by Pawłowski and Staszak (2016), during seed dormancy release, MDH levels could also be regulated via endogenously applied gibberellin and abscisic acid. Notably, in the analysis by Stavrinides et al. (2019), desiccation-tolerant seeds of *Coffea arabica* and *Coffea eugenioides* exhibited a downregulation of *MDH* gene expression, aligning with the outcomes of our proteomic analyses.

Another protein identified in our study in recalcitrant sycamore seed axes as part of the TCA cycle is aconitate hydratase. This enzyme plays a crucial role in catalyzing the conversion of citrate to isocitrate, and it is one of the susceptible oxidation-sensitive enzymes in the TCA cycle (Lushchak et al., 2014). Notably, our findings indicate the potential redox regulation of this protein by Trxs. Aconitate hydratase has been proposed to function as a redox sensor within the TCA cycle. Its sensitivity to reactive oxygen and nitrogen species is attributed to the inherent instability of its [4Fe–4S]2+ prosthetic group, leading to the formation of an inactive [3Fe–4S]+ cluster upon oxidation (Robbins and Stout, 1989; Beinert et al., 1996; Mansilla et al., 2023).

Other proteins involved in the TCA cycle identified as potential targets of Trxs were methylmalonate-semialdehyde dehydrogenase (MMSDH) and oxoglutarate dehydrogenase. MMSDH is an enzyme located in mitochondria and is part of the distal section of the inositols pathway. Its function includes converting malonate and methylmalonate-semialdehydes into acetyl-CoA through oxidative decarboxylation (Ferreira-Neto et al., 2021). *Arabidopsis* plants with a knockout of *MMSDH* produced seeds with wrinkled coats, reduced storage reserves, increased valine and leucine concentrations, and lower germination rates (Gipson et al., 2017). It is known that oxoglutarate dehydrogenase (OGDH) in *Arabidopsis* occurs in isoforms, which influence the development of various plant organs. In the study of *Arabidopsis* E1 subunit of the 2-OGDH role in plant and seed development it has been revealed that the *e1-ogdh*2 mutant lines showed an interesting phenotype with regard to reserve accumulation during seed development, resulting in increased seed weight and total seed weight per plant. Moreover, there was a reduction in the number of seeds per silique in the *e1-ogdh* mutant lines (Condori-Apfata et al., 2019). In our study, proteins of the TCA cycle were identified as targets of Trxs exclusively in recalcitrant seeds, whereas we did not observe such interactions in orthodox seeds. This leaves the question of whether TCA-associated proteins influence the lack of tolerance to desiccation in recalcitrant seeds.

The shared proteins identified in orthodox Norway maple and recalcitrant sycamore seeds, namely, glutathione peroxidase, glutathione transferase, glyceraldehyde-3-phosphate dehydrogenase, phosphoglucomutase, and nucleoside diphosphate kinase, are involved in a diverse array of processes, including ROS scavenging (Navrot et al., 2006; Singh and Reindl, 2021; Bela et al., 2022), carbohydrate metabolism (Manjunath et al., 1998; Periappuram et al., 2000; Michelet et al., 2013; Zaffagnini et al., 2013, 2013), and nucleoside triphosphate synthesis (Bernard et al., 2000; Dorion and Rivoal, 2018; Lu and Hunter, 2018; Wolfe et al., 2019; Georgescauld et al., 2020).

The identification of a limited number of proteins associated with diverse metabolic processes in orthodox seeds corresponds with previous proteomic and transcriptomic investigations, indicating that during the maturation of orthodox seeds, they coordinately downregulate their metabolism (Leprince et al., 1999; Choi et al., 2014; Wang et al., 2015; Stavrinides et al., 2019; Kijak and Ratajczak, 2020). As a result, they become tolerant to desiccation, enabling prolonged storage under controlled conditions.

#### 3.2.2 Genetic information processing

Within the subset of proteins associated with genetic information processing, 78 proteins were identified in recalcitrant seeds, while 33 were found in orthodox seeds. Only eight proteins were common to both seed types (**Figure 6**, **Table 1**, **Supplementary Table 3**).

Upon comparing the functions of proteins identified in recalcitrant seeds to those specific to orthodox seeds, it appears that proteins involved in stress response, protein folding, and nucleic acid regulation are more dominant in recalcitrant seeds. Proteins such as heat shock proteins (HSPs), chaperonins, and glutaredoxin-dependent peroxiredoxins, which are involved in the stress response (Laporte et al., 2012; Gomez-Pastor et al., 2018; Roy et al., 2019; Bourgine and Guihur, 2021), were identified in recalcitrant seeds. These proteins likely play a crucial role in maintaining cellular homeostasis and protecting seed integrity under stress conditions. Furthermore, chaperonins, such as chaperonin CPN60–2 and the TCP-1/cpn60 chaperonin family protein, were identified in recalcitrant seeds. Chaperonins assist in protein folding and assembly and prevent protein aggregation (reviewed in Saibil, 2013). The presence of these proteins suggests the importance of proper protein folding and protein conformation maintenance in recalcitrant seeds, potentially due to the need for proteins to withstand stress and aging during storage.

While protein synthesis, modification, and degradation are common functions shared by both orthodox and recalcitrant seeds, the dominance of stress response proteins, chaperonins, and nucleic acid regulation proteins in recalcitrant seeds may be associated with adaptive mechanisms to facilitate germination while skipping the long dormancy period (Duncan et al., 2019).

Among the axes of orthodox Norway maple seeds, we identified ribosomal proteins (e.g., 40S ribosomal protein S8 and 60S acidic ribosomal proteins), elongation factors (e.g., elongation factor 1-alpha, elongation factor 1-gamma-like eukaryotic, translation initiation factor 5A), and initiation factors (e.g., eukaryotic initiation factor 4A-11), among others. These proteins play a crucial role in ensuring the accurate and efficient translation of mRNAs into proteins (Svitkin et al., 2001; Tariq et al., 2016; Martinez-Seidel et al., 2020; Xu et al., 2023).

#### 3.2.3 Signaling and cellular processes

In this category, we identified 18 proteins in recalcitrant seeds and four in orthodox seeds, two of which were shared by both seed categories (**Figure 7**, **Table 1**, **Supplementary Table 3**). Among the shared proteins, we identified actin, with confirmed oxidation of all six cysteines in β/γ-actin and five cysteines in α-actin (Balta et al., 2021).

Research findings suggest that the Trx protein plays a role as a signaling component during the initial stages of seed germination. Trx plays a key role in facilitating the mobilization of seed reserves by reducing seed storage proteins (SSPs), thereby increasing their solubility and susceptibility to proteolytic degradation (Yano et al., 2001; Wong et al., 2004a; Shahpiri et al., 2008). Consequently, the presence of proteases in the proteomes (**Supplementary Table 3**) of both examined seed types aligns with expectations. Their functionality and regulation by Trxs are likely to be important for seeds, irrespective of their desiccation resistance level.

#### 3.2.4 Environmental information processing

Among the identified proteins, we observed members of the environmental information processing protein group. A notable difference in the number of identified proteins between Norway maple (1) and sycamore (13) seeds was observed (**Supplementary Table 3**). Only one protein, superoxide dismutase, was identified in Norway maple seeds.

Among the proteins identified in sycamore seed axes were calmodulin-binding protein (CBP). CBPs are known for their involvement in morphogenesis, cell division, cell elongation, ion transport, gene regulation, cytoskeletal organization, cytoplasmic streaming, pollen function, and stress tolerance (Snedden and Fromm, 1998; Zielinski, 1998; Reddy, 2001). Another crucial protein identified was the guanine nucleotide-binding protein (G protein). Heterotrimeric G-proteins are essential for signal transduction and regulate plant responses to both biotic and abiotic stresses (Gao et al., 2019; Patel et al., 2020; Urano et al., 2020; Ninh et al., 2021). L-ascorbate oxidase, an enzyme from the blue copper oxidase family, exhibits peak activity in regions of cell expansion, suggesting it may influence cell expansion and modulate hormone and redox signaling in the apoplast (Esaka et al., 1992; Pignocchi and Foyer, 2003; Pignocchi et al., 2006; De Tullio et al., 2013; Smirnoff, 2018). Moreover, this enzyme plays a crucial role in stress response mechanisms (Pignocchi et al., 2006; Garchery et al., 2013). Profilins, small actin-binding proteins found in eukaryotes and certain viruses, are crucial for cell development, cytokinesis, membrane trafficking, and cell motility (Krishnan and Moens, 2009). AtPFN1 and AtPFN2, isolated from *Arabidopsis* phloem sap, have been shown to possess antifungal effects by inducing apoptosis, involving membrane potential breakdown and the release of cytochrome C within fungal cells (Christensen et al., 1996; Huang et al., 1996; Park et al., 2018; Son et al., 2022).

The functions of four identified proteins in this category: alcohol dehydrogenase-like protein 7 (dehydrin), desiccation-related protein PCC3–06-like (LEA), low-temperature-induced 65 kDa protein, and vicilin-like antimicrobial peptide A, have not yet been described with specific functions in the scientific literature to date.

Four records that corresponded to specific domains, will be not discussed as domains because they may be part of many proteins with various functions.

Special attention was given to superoxide dismutase (SOD) activity observed in both *Acer* species. Confirmation was achieved through the analysis of native PAGE SOD isoforms, which indicated that it was a Cu/Zn-SOD present in both fresh and stored seeds of Norway maple and sycamore (**Supplementary Figure 2**). Like other SODs, Cu/Zn SODs catalyze the dismutation of two superoxide radicals (O_2_^.-^) and water into hydrogen peroxide (H_2_O_2_) and O_2_, with H_2_O_2_ levels tightly regulated by the Trx system (Foyer and Hanke, 2022; Sevilla et al., 2023). It possesses a Cu(II) plus Zn(II) active site and is found in the cytosol and plastids. Along with ascorbate peroxidase, Cu/Zn-SOD is the first line of defense against oxidative stress (Lee et al., 2010).

Our previous studies indicated that the levels of O_2_. and H_2_O_2_ in Norway maple seeds peak between 16th and 18th WAF, coinciding with the acquisition of desiccation tolerance. In sycamore seeds, O_2_ **^.-^** levels are significantly elevated in the early stages of development, while H_2_O_2_ levels remain low during maturation and in mature seeds (Ratajczak et al., 2019b). We hypothesize that O_2_ **^.-^** may participate in regulating metabolic processes in Norway maple seeds associated with desiccation tolerance acquisition (Ratajczak et al., 2019b). The presence of Cu/Zn-SOD, a target protein of Trxs, in both species indicates that the redox regulation associated with ROS levels in mature seeds is active, metabolic activity is at a normal level, and tolerance to oxidative stress may be increased (Lee et al., 2010). Considering not only the obtained results but also our earlier data (Pukacka and Ratajczak, 2007a; Ratajczak et al., 2019b), it is plausible that in the case of Norway maple, this mechanism is linked to the acquisition of desiccation tolerance, potentially increasing seed viability during storage. In sycamore, this mechanism may guarantee increased seed viability through increased tolerance to oxidative stress.

In summary, orthodox seeds demonstrate a more effective shutdown of their metabolism, contributing to their prolonged viability. In contrast, recalcitrant seeds maintain a basal metabolic activity, which is evident from the number of targets identified in this study. Consequently, the increased number of Trx targets in recalcitrant seeds suggests partial metabolic activity during storage. This partial activity could provide an advantage in terms of rapid germination and seedling establishment, as these seeds can reactivate more quickly than others. However, this ongoing metabolic activity may also lead to reduced long-term viability due to continuous energy consumption and depletion of metabolites.

## 4 Conclusions

Our findings revealed significant differences in transcript levels between recalcitrant and orthodox seeds, underscoring the critical role of redox regulation in seed development, desiccation tolerance, and storage. Proteomic analysis identified numerous Trx-h1 target proteins, with distinct profiles observed in each seed category. Recalcitrant seeds exhibited a diverse array of proteins related to metabolism, stress response, and signaling, suggesting specialized adaptive mechanisms to counteract storage-related stresses. In contrast, orthodox seeds displayed a conserved protein profile, indicative of robust molecular processes that facilitate germination and seedling growth. Overall, the results of this study highlight the crucial role of redox regulation in seed biology and lay the groundwork for future research focusing on adaptations to a changing climate. As the latter is one of the main challenges in biodiversity conservation and sustainable forest management, these results are crucial for maintaining the persistence of the studied species under a changing climate.

## Data availability statement

All relevant data is contained within the article: The original contributions presented in the study are included in the article/**Supplementary Material**, further inquiries can be directed to the corresponding author/s.

## Author contributions

HF: Conceptualization, Data curation, Formal analysis, Writing – original draft, Writing – review & editing. AMS: Data curation, Methodology, Writing – review & editing. PV: Formal analysis, Methodology, Writing – review & editing. MS: Methodology, Writing – review & editing. AJS: Data curation, Formal analysis, Methodology, Writing – review & editing. MD: Writing – review & editing. EK: Formal analysis, Writing – review & editing. PG: Data curation, Formal analysis, Writing – review & editing. KR: Writing – review & editing. ER: Conceptualization, Data curation, Formal analysis, Funding acquisition, Investigation, Methodology, Project administration, Writing – review & editing.
