# Supplementary material for: Redox dynamics in seeds of Acer spp: unraveling adaptation strategies of different seed categories
Source: Front Plant Sci. 2024 Jul 24;15:1430695. doi: 10.3389/fpls.2024.1430695 (PMC11303208; doi:10.3389/fpls.2024.1430695)
Supplement: Supplementary file 1 [file DataSheet_1.docx]

**Supplementary Figures**

**Redox dynamics in seeds of *Acer* spp: unraveling adaptation strategies of different seed categories**

Hanna Fuchs, Aleksandra M. Staszak, Paola A. Vargas, Mariam Sahrawy, Antonio J. Serrato, Marcin K. Dyderski, Ewelina A. Klupczyńska, Paweł Głodowicz, Katarzyna Rolle, Ewelina Ratajczak

**
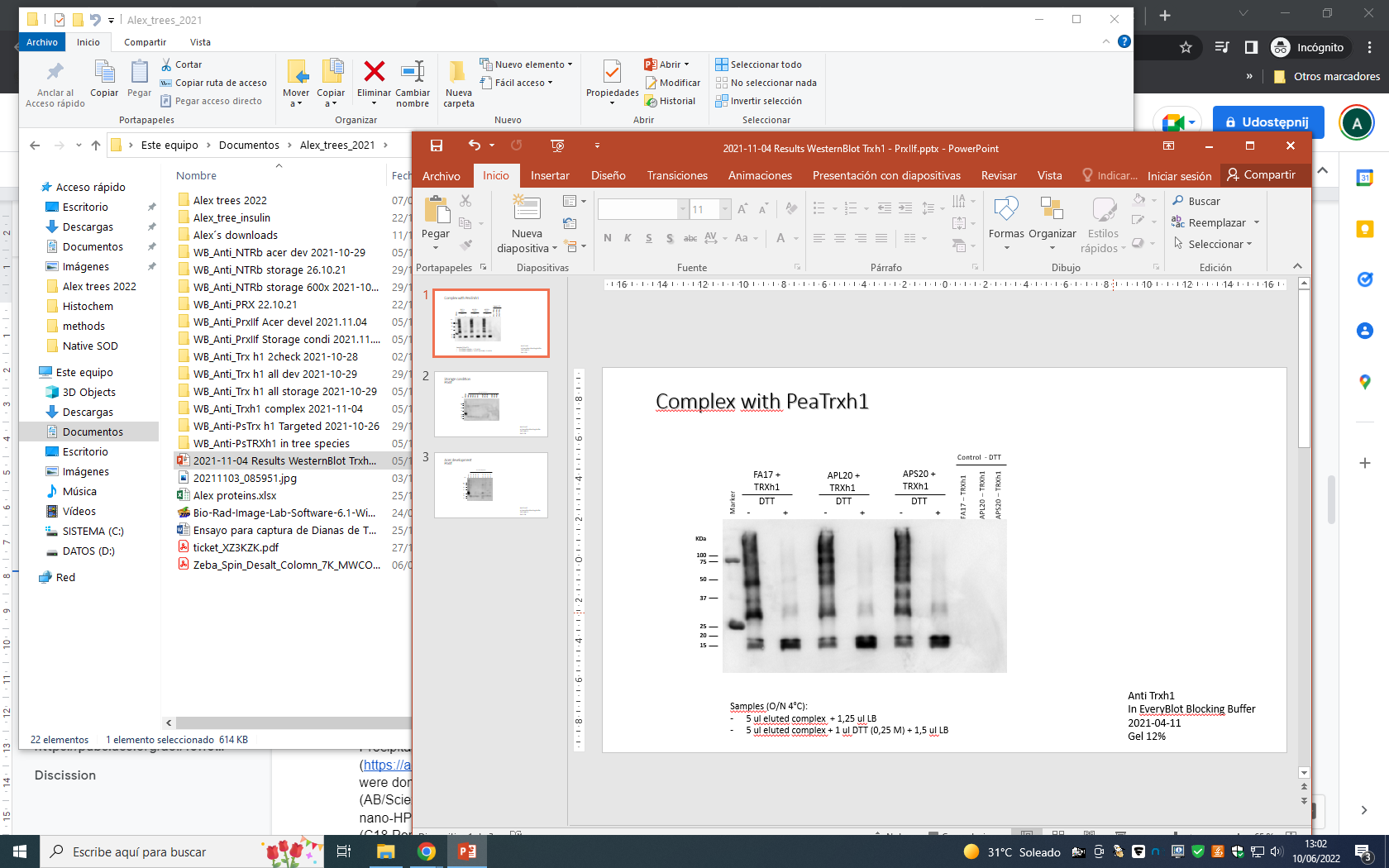
**

SF 1. Western Blotting of Trx-h1 targets Norway maple (APL), sycamore (APS) without DTT we see complexes with Trx-h1, dimer and monomer of Trx-h1, the control trial without Trx-h1 and DTT is shown in the last three lines.


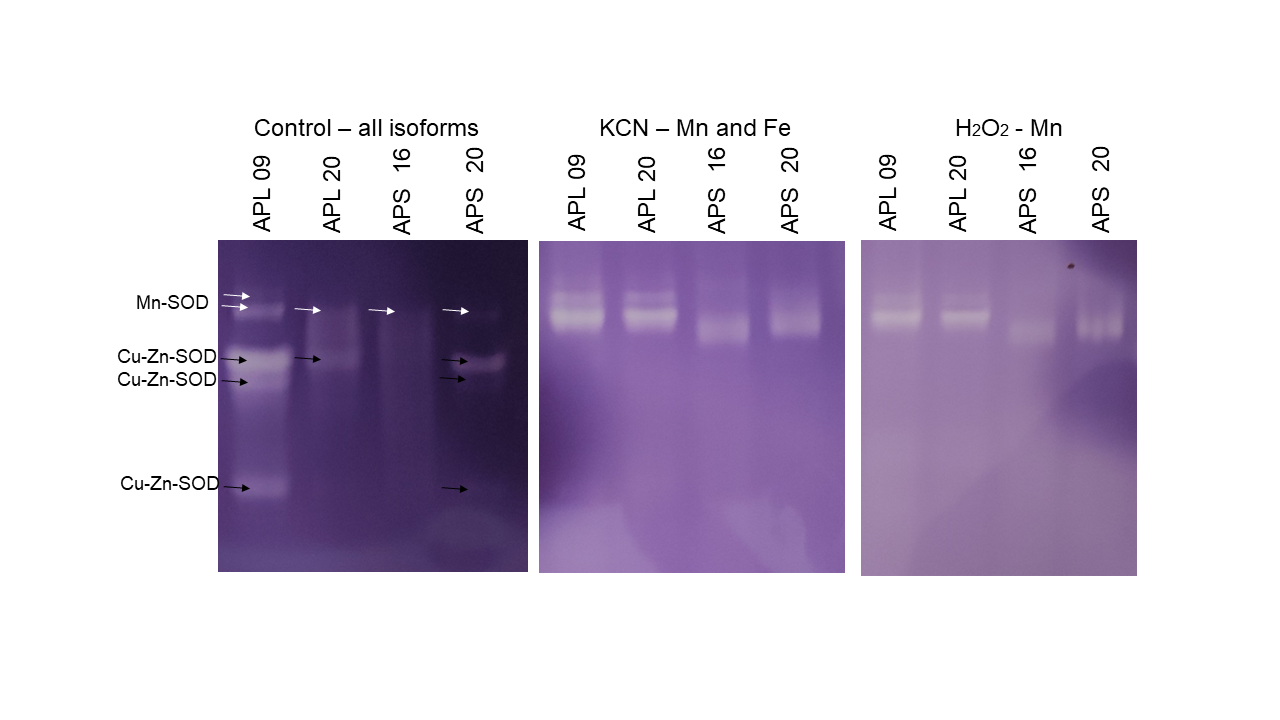


SF 2.Analysis of native PAGE SOD isoforms. Norway maple (APL), sycamore (APS), the number accompanying the sample name indicates the year of collection.. During seed storage in both Acer species, the activity of superoxide dismutase (SOD) decreased. We identified two forms of SOD: Cu-Zn-SOD and Mn-SOD. The SOD activity was lower in the stored for a shorten time. SOD isoforms were detected according to Beauchamp and Fridovich (1971).

**Reference:**

Beauchamp, C. and Fridovich, I. 1971. Superoxide Dismutase: Improved Assays and an Assay Applicable to Acrylamide Gels. Anal. Biochem., 44: 276–287.
