# Supplementary material for: Redox dynamics in seeds of Acer spp: unraveling adaptation strategies of different seed categories
Source: Front Plant Sci. 2024 Jul 24;15:1430695. doi: 10.3389/fpls.2024.1430695 (PMC11303208; doi:10.3389/fpls.2024.1430695)
Supplement: Supplementary file 2 [file Table_1.docx]

**Supplementary Table 1**

Redox dynamics in seeds of *Acer* spp: unraveling adaptation strategies of different seed categories

Hanna Fuchs, Aleksandra M. Staszak, Paola A. Vargas, Mariam Sahrawy, Antonio J. Serrato, Marcin K. Dyderski, Ewelina A. Klupczyńska, Paweł Głodowicz, Katarzyna Rolle, Ewelina Ratajczak

Table S3 Primer sequences used in the qPCR experiment

|  | **Target gene** | **Oligonucleotide name** | **Sequence 5’-3’** | **Product size** bp | **SELF** | **Locus tag NCBI** | **Reference sequence**  **BLAST NCBI** | **Sequence ID BLAST** |
| --- | --- | --- | --- | --- | --- | --- | --- | --- |
| 1 | Thioredoxin H-type 1 | **TRX1_F** | CCTGTATCGCCCAATCACTT | 196 | 1/0 | AT3G51030 | Chromosome: 3 NC_003074.8 | **LR782544.1** |
| 2 |  | **TRX1_R** | CGTTTCATCGCTCCATTCTT | 196 | 1/0 | AT3G51030 | Chromosome: 3 NC_003074.8 | **LR782544.1** |
| 3 | NADPH-dependent thioredoxin reductase A | **TrxR_NTRA _F** | TGGTGGTGGTGATTCAGCTA | 181 | 3/0 | AT2G17420 | Chromosome: 2 NC_003071.7 | **LR782543.1** |
| 4 |  | **TrxR_NTRA_R** | TTCATCACCATACGCCTCAA | 181 | 3/0 | AT2G17420 | Chromosome: 2 NC_003071.7 | **LR782543.1** |
| 5 | 1-cysteine peroxiredoxin | **1-Cys Prx_F** | TTCCCACAGGGTTTCAAGAC | 193 | 3/2 | AT1G48130 | Chromosome: 1 NC_003070.9 | **LR782542.1** |
| 6 |  | **1-Cys Prx_R** | GTGCACCACATCATCACCAT | 193 | 3/2 | AT1G48130 | Chromosome: 1 NC_003070.9 | **LR782542.1** |
| 7 | Elongation factor 1 alpha  (GTP binding Elongation factor Tu family protein) | **EF1a_F** | CCGTTCCAATACCACCAATC | 167 | 0/1 | AT1G07940 | Chromosome: 1  NC_003070.9 | **CP002684.1** |
| 8 |  | **EF1a_R** | TGGATTTGAGGGTGACAACA | 167 | 0/1 | AT1G07940 | Chromosome: 1  NC_003070.9 | **CP002684.1** |
